# The potential of bamboo seeds for natural biofortification of dietary zinc and iron

**DOI:** 10.1038/s41538-023-00192-4

**Published:** 2023-04-21

**Authors:** Qifang Hu, Rong Wang, Lin Hu, Rong Chen, Xuejun Yu, Ji Feng Shao

**Affiliations:** 1 State Key Laboratory of Subtropical Silviculture, Zhejiang Agriculture & Forestry University, Lin’An, 311300 China; 2 Marketing supervision administration of Jiande, Jiande, 311612 China

**Keywords:** Plant sciences, Agriculture

## Abstract

Moso bamboo has been shown to accumulate high concentrations of iron and zinc in the seeds. However, the bioavailablity of iron and zinc in bamboo seeds is poorly understood. Here, we evaluated the bioaccessibility and bioavailability of iron and zinc in bamboo seeds by using an in vitro digestion protocol. Our evaluations revealed that values of bioaccessibility and bioavailability of iron were 25 and 21 mg kg^−1^ in bamboo seeds which were 1.6- and 1.7- fold higher than in rice, respectively. Also, values of bioaccessibility and bioavailability of zinc were 20 and 13 mg kg^−1^ in bamboo seeds which were 1.9- and 2.6- fold higher than in rice, respectively. Boiling process reduced both the bioaccessibility and bioavailability of iron and zinc. In addition, phytic acid concentration in bamboo seeds was only 0.42 times higher than in rice. By contrast, the tannins concentration in bamboo seeds was 2.2 times higher than in rice. Cellular localization results showed that iron and zinc were mainly concentrated in the embryo and the aleurone layer. These results clearly suggest that Moso bamboo seeds are rich in iron and zinc and have potential as a food for iron and zinc biofortification.

## Introduction

Iron (Fe) and Zinc (Zn) are two micronutrients that play an important role in sustaining healthy metabolic activities^1^. For example, Fe is well known as an essential component of hemoglobin and it is responsible for transporting oxygen, while Zn acts as catalytic factor regulating enzymatic activity^2,3^. However, both Fe and Zn deficiency result in public health problems^4–6^. It is reported clinically certain lower concentration of Fe cause Fe-deficiency anemia and also lower concentration of Zn impact on impaired immunity, impaired neurodevelopment, and stunted growth^7–9^. More than two billion people are estimated to be suffering Fe deficiency and billons of people are at risk of Zn deficiency^10,11^. The major group of people with Fe and Zn deficiency are preschool-aged children and pregnant women, and most of these people live in low-income regions or in developing countries^12–14^, such as eastern Africa and south-eastern Asia^5,14^. It is reported in a recent nutrition survey in Pakistan that above 18% of preschool-aged children and more than 20% of women at reproductive age suffers from lower concentration of Zn^15^. Also, in China, Fe and Zn malnutrition remain widely prevalent problems, and approximately 245 and 100 million of people are suffering by low concentration of Fe and Zn, respectively, with most of them living in rural areas^10^.

To this point, the management and prevention of Fe and Zn malnutrition have thus been receiving considerable attention worldwide. Many efforts and strategies have been investigated to overcome the above problems, including: (i) Direct Fe and Zn supplementation, such as administration of zinc or iron salt tablets (e.g., gluconates) under a doctor’s advice^16–18^. (ii) Fe and Zn food biofortification, such as plant breeding techniques, foliar mineral fortification, and genetic engineering where the concentration of Fe and Zn in crop foods can be increased^12,19^. (iii) Increasing Fe and Zn bioavailability by reducing the concentrations of anti-nutrients. The biofortification of Fe and Zn in crop foods does not ensure Fe and Zn bioavailability because of the presence of nutrient inhibitors, such as phytate and tannins. Therefore, decreasing the concentrations of nutrient inhibitors, such as phytate, has been attempted. By interfering with phytate biosynthesis in plants, the phytate levels in grains is reported to be decreased and the bioavailability of Fe and Zn was significantly increased^16^. (iv) Dietary diversification is another effort in which by increasing variety of foods instead of consuming non-diversified cereal and plant-based diets^10–17^.

Bamboo seed, caryopsis of bamboo, is a rare and precious food which is the only food for the divine bird, the phoenix, according to an ancient Chinese legend. It is rare to see bamboo seeds because most bamboo species only seed after a long vegetative growth period^20,21^. However, in some Asian regions, such as southwest China and southern India, bamboo seeds are an important food resources^22,23^. It is reported that bamboo seeds are also used in folk medicines and in therapeutic applications in astringent or hypolipidemic, as well as in polycystic ovarian disease^21^. As a good substitute for rice, bamboo seeds are considered as food resource for dietary diversity because of the high nutritional value of the seeds, and many studies have investigated bamboo seeds.

In our previous study, we investigated the profile of mineral elements in Moso bamboo seeds as one of the most widely distributed and cultivated bamboo species in China and found that the concentrations of Fe and Zn in bamboo seeds were very high^23^. To confirm if high Fe and Zn accumulation in Moso bamboo seeds is universal in nature, it is better to investigate Fe and Zn concentration in Moso bamboo seeds harvest from different geographical sites with different habitations. Besides, there is lack of information such as how is the bioaccessibility and bioavailability of Fe and Zn in Moso bamboo seeds, where is the Fe and Zn main deposited in Moso bamboo seeds, and so on. Therefore, the objectives of present study were (i) to confirm Fe and Zn concentration in bamboo seeds; (ii) to evaluate the bioaccessibility and bioavailability of Fe and Zn in bamboo seeds; and (iii) to determine Fe and Zn cellular localization in bamboo seeds. We found that bamboo seeds are rich in Fe and Zn, which indicated the potential of bamboo seeds as a bio-diverse food for natural Fe and Zn biofortification.

## Results

### Fe and Zn concentrations in Moso bamboo seeds

**Fig. 1:**
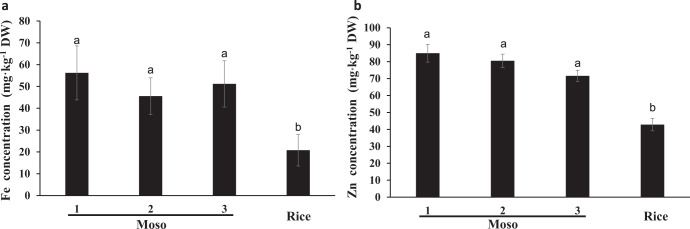
Fe and Zn concentration in Moso bamboo seeds and rice. **a** Fe concentration in Moso bamboo seeds and rice. **b** Zn concentration in Moso bamboo seeds and rice. Seeds of Moso bamboo collected from three different geographical sits were named 1, 2, and 3, respectively. The concentration of Fe and Zn in bamboo seeds and rice was determined by ICP-MS. Data are means ± SD (*n* = 3). Statistical comparison was performed by one-way ANOVA, followed by Tukey’s multiple comparison test. Different lower-case letters indicate significant difference at *P* < 0.05.

We collected Moso bamboo seeds from three different geographical sites and compared the Fe and Zn concentrations in Moso bamboo seeds with those in rice. Our results showed that the Fe concentration in Moso bamboo seeds was much higher than in rice (Fig. 1a). The Fe concentration in bamboo seeds were collected from Haiyang (site 1) reached 56.2 mg kg^−1^, which was 2.5-fold higher than the concentration in rice. Also, Zn concentration in Moso bamboo seeds was significantly higher than in rice (*P* < 0.05). The concentrations of Zn in bamboo seeds were 84.9, 80.5, and 71.6 mg kg^−1^ in seeds collected from the three different places, which were almost two-fold higher than that reported in rice (Fig. 1b).

### Bioaccessibility and bioavailability of Fe and Zn in Moso bamboo seeds

**Fig. 2:**
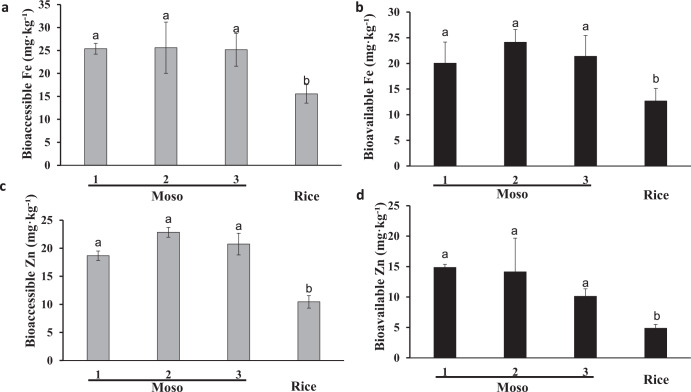
Bioaccessible and bioavailable of Fe and Zn concentration in Moso bamboo seeds and rice. **a** Bioaccessible of Fe concentration in Moso bamboo seeds and rice. **b** Bioavailable of Fe concentration in Moso bamboo seeds and rice. **c** Bioaccessible of Zn concentration in Moso bamboo seeds and rice. **d** Bioavailable of Zn concentration in Moso bamboo seeds and rice. Seeds of bamboo and rice were simulated a gastrointestinal digestion in vitro, the Fe and Zn concentration in each fraction was determined by ICP-MS. Data are means ± SD (*n* = 3). Statistical comparison was performed by one-way ANOVA, followed by Tukey’s multiple comparison test. Different lower-case letters indicate significant difference at *P* < 0.05.

High concentration of Fe and Zn does not always equate to high bioavailability of Fe and Zn, because they are not in good correlation between the concentrations of nutrient minerals in raw grains and their bioavailability^24^. On the other hand, different cooking processes have different effects on the bioaccessibility and bioavailability of Fe and Zn in seeds^19,25^. Therefore, we evaluated the bioaccessibility and bioavailability of Fe and Zn in both raw and boiled Moso bamboo seeds. The Fe bioaccessibility in Moso bamboo seeds was 25.3, 25.6, and 25.1 mg kg^−1^ collected form Haiyang County (site 1), Fusui County (site 2), and Lingchuan County (site 3), respectively, which are higher than those values reported in rice (Fig. 2a). Moso bamboo seeds collected from Fusui County (site 2) had almost two-fold higher bioaccessibility than rice. After boiling, the Fe bioaccessibility was decreased for all the seed samples, but the bioaccessibility of Fe in bamboo seeds were still significantly higher than the values in rice (Fig. S1a). Similar trends were also found in Fe bioavailability data. Figure 2b shows that Fe bioavailability in Moso bamboo seeds was significantly higher than those values reported in rice (*P* < 0.05). While boiling also decreasing the Fe bioavailability in all seeds, but the bioavailability of Fe in bamboo seeds were much higher than in rice as well (Fig. S1b).

We observed that Zn bioaccessibility and bioavailability in all the Moso bamboo seed samples were significantly higher than those values reported in rice (Fig. 2c, d). Boiling process also effect the bioaccessibility and bioavailability of Zn in bamboo seeds and rice. However, the bioaccessibility and bioavailability of Zn in Moso bamboo seeds were still higher than in rice (Fig. S1c, d).

### Phytic acid (PA) and tannins concentrations in Moso bamboo seeds

**Fig. 3:**
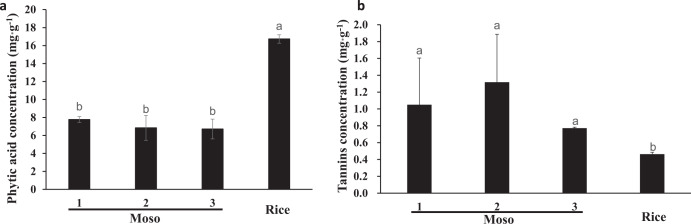
Phytic acid and tannins concentration in Moso bamboo seeds and rice. **a** Phytic acid concentration in Moso bamboo seeds and rice. **b** Tannins concentration in Moso bamboo seeds and rice. Data are means ± SD (*n* = 4). Statistical comparison was performed by one-way ANOVA, followed by Tukey’s multiple comparison test. Different lower-case letters indicate significant difference at *P* < 0.05.

Many anti-nutritional factors have a great influence on the mineral bioavailability. For example, PA and tannins have been described as inhibitors of Fe and Zn. Therefore, in our study, we investigated the presence of PA concentration in Moso bamboo seeds and rice. The PA concentrations in the seeds of Moso bamboo collected from different places were 7.77, 6.84, and 6.71 mg g^−1^, which were much lower than those values reported in rice (Fig. 3a). We further calculated the molar ratios of phytate to Fe and phytate to Zn in both bamboo seeds and rice (Table 1). The molar ratios of phytate to Fe were 11:1, 12:1, and 11:1 in bamboo seeds, which were much lower than those values reported in rice (68:1). The molar ratios of phytate to Zn were 9:1, 8:1, and 9:1, which were also much lower than those values reported in rice (Table 1). These results clearly indicated that Moso bamboo seeds have a low concentration of PA, and PA is probably not the key inhibitory factor that may influences bioaccessibility and bioavailability of Fe and Zn in Moso bamboo seeds.

**Table 1:** Fe Zn and phytic acid (PA) concentration of Moso bamboo seeds and rice.

Sample number		Fe(mg·kg^−1^)	Zn(mg·kg^−1^)	Phytic Acid (g·kg^−1^)	Molar Ratio PA:Fe	Molar Ratio PA:Zn
Moso	1	56.2a	85.0a	7.8b	11:1	9:1
	2	45.6a	80.5a	6.8b	12:1	8:1
	3	51.2a	71.7a	6.7b	11:1	9:1
Rice		20.8b	42.8b	16.7a	68:1	38:1

Data are means ± SD (*n* = 3). Statistical comparison was performed by one-way ANOVA, followed by Tukey’s multiple comparison test. Different lower-case letters indicate significant difference at *P* < 0.05.

Tannins is one of the component in many plant foods and can act as a nutrient inhibitor by forming insoluble complexes with nutrients. In our results, we found that tannins concentration in bamboo seeds was much higher than in rice. As seen in Fig. 3b, their values are 1.05, 1.31, and 0.77 mg g^−1^ in Moso bamboo seeds in comparison with rice which is reported only 0.46 mg g^−1^ (Fig. 3b). We further determined that PA and tannins concentrations after boiling. We found that bamboo seeds contained a lower PA concentration than that in rice, but the concentration of tannins in bamboo seeds was much higher than that in rice after boiling (Fig. S2). However, both PA and tannins concentrations were decreased after boiling, which indicated that PA and tannins were degraded. These results suggested that high concentration of tannins in Moso bamboo seeds may be a key anti-nutritional factor, which considerably affected Fe and Zn bioaccessibility and bioavailability.

### Cellular localization of Fe and Zn in Moso bamboo seeds

**Fig. 4:**
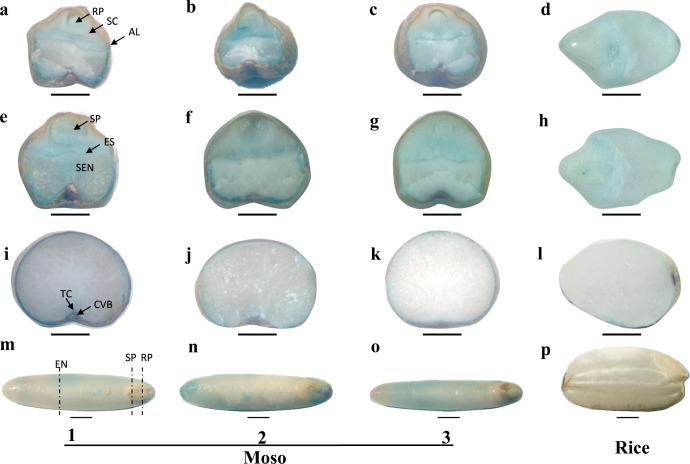
Localisation of Fe in Moso bamboo seeds and rice. Fe in transverse sections of root primordium (**a**, **b**, **c**, **d**), shoot primordium (**e**, **f**, **g**, **h**), and endosperm (**i**, **j**, **k**, **l**) of Moso bamboo seeds (**a**, **b**, **c**, **e**, **f**, **g**, **i**, **j**, **k**) and rice (**d**, **h**, **l**) were stained by Prussian blue staining. RP root primordium; SP shoot primordium; EN endosperm; SEN starchy endosperm; SC scutellum; ES epithelium of scutellum; AL aleurone layer; TC transfer cell; CVB crease vascular bundle. Scale bars = 1000 μM.

**Fig. 5:**
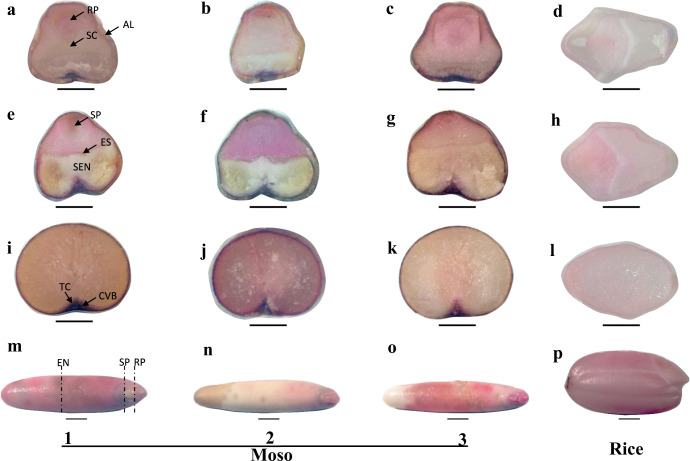
Localisation of Zn in Moso bamboo seeds and rice. Zn in transverse sections of root primordium (**a**, **b**, **c**, **d**), shoot primordium (**e**, **f**, **g**, **h**), and endosperm (**i**, **j**, **k**, **l**) of Moso bamboo seeds (**a**, **b**, **c**, **e**, **f**, **g**, **i**, **j**, **k**) and rice (**d**, **h**, **l**) were stained by dithizone staining. RP root primordium; SP shoot primordium; EN endosperm; SEN starchy endosperm; SC scutellum; ES epithelium of scutellum; AL aleurone layer; TC transfer cell; CVB crease vascular bundle. Scale bars = 1000 μM.

The cellular localization of Fe and Zn was investigated by Prussian blue staining and dithizone staining in Moso bamboo seeds. Three transverse sections of seeds were prepared using a slicer (Fig. 4). Fe and Zn were mainly concentrated in the embryo and the aleurone layer of the endosperm; by contrast, the starchy endosperm accumulated much less Fe and Zn (Figs. 4 and 5). In the section of root primordium, Fe was predominantly concentrated in the scutellum of the embryo and the aleurone layer of the endosperm, while in shoot primordium section, Fe was mainly localized in the epithelium of the scutellum and the aleurone layer. In the endosperm transverse section, Fe was mainly concentrated in the outer layer of the endosperm. We also found that Zn was predominantly concentrated in the scutellum of the embryo and the aleurone layer of the endosperm in the root primordium section. In shoot primordium and endosperm sections, Zn was also accumulated in the aleurone layer of the endosperm, but different from the Fe localization, Zn was also highly accumulated in the groove region where the transfer cell and crease vascular bundle are located (Fig. 5). A comparison of the pictures indicated that the blue and red colored stain in three different seeds showed some difference in the color depth, but their localization was similar. All the bamboo seeds appeared to be more highly stained with the blue and red colored stains than rice, which was consistent with the data that Moso bamboo seeds contained higher Fe and Zn concentrations than rice seeds.

We further mapped Fe and Zn deposition in Moso bamboo seeds by LA–ICP–MS in both longitudinal and transverse sections. The map of elemental carbon cellular distribution was used as a control (Fig. S3e, f). Our results showed that Fe was mainly distributed in the aleurone layer of the bamboo seeds in longitudinal sections (Fig. S3a). For the transverse sections, we found that Fe not only was highly distributed in pericarp (aleurone layer), but also it was highly accumulated in the embryo (Fig. S3b). These results were consistent with the results for the Prussian blue staining shown in Fe earlier. Also, for Zn, a similar cellular distribution pattern was found in both longitudinal and transverse sections and signal for Zn was also high in pericarp and embryo (Fig. S3c, d). In contrast, carbon was dispersed homogeneously in both longitudinal and transverse sections (Fig. S3e, f).

## Discussion

To deal with limited Fe/Zn and improve Fe/Zn resources nutritional conditions, several attempts are being considered worldwide. Increasing in food resources are among of those attempts to increase Fe and Zn intake in humans^14^. However, in many underdeveloped regions, where cereal-based foods are the staple food in the daily diet, food diversity is difficult to achieve^10^. Therefore, increasing Fe and Zn concentrations in cereals, or the discovery of other starchy staples rich in Fe and Zn, is most applicable. In the present study, we found that Moso bamboo naturally contains higher concentrations of Fe and Zn in the seeds, in comparison with the standard crop cereal, rice. For instance, it is reported that in rice, Fe concentration ranged from 7.5 to 24.4 mg kg^−1^ with a mean value of 12.1 mg kg^−1^ and the Zn concentration ranged from 15.9 to 58.4 mg kg^−1^ with a mean of 25.4 mg kg^−1^ ^26^. In maize, Fe and Zn concentrations have been shown to range from 16.4 to 22.9 mg kg^−1^ (mean 19.6 mg kg^−1^) and 14.7 to 24.0 mg kg^−1^ (mean 19.8 mg kg^−1^), respectively^27^. Wheat contains 28.8 to 56.5 mg kg^−1^ of Fe with a mean value of 37.2 mg kg^−1^, and 25.2 to 53.3 mg kg^−1^ of Zn with a mean value of 35.0 mg kg^−1^ ^26^. In Moso bamboo seeds, Fe concentration ranged from 45.5 to 56.2 mg kg^−1^ with a mean of 50.9 mg kg^−1^, and Zn concentration ranged from 71.6 to 84.9 mg kg^−1^ with a mean value of 79.0 mg kg^−1^. Both Fe and Zn concentrations were much higher in bamboo seeds in comparison with those values reported in rice, maize, or wheat, indicating that Moso bamboo seeds may have potential value as an available resource for Fe and Zn biofortification.

According to the nutrient intake reference of the World Health Organization and Food and Agriculture Organization (2004)^28^, the recommended daily dietary requirement for Fe is 13 and 29 mg/day for men and women, respectively, and for Zn is 14 and 10 mg/day for men and women, respectively^14^. Based on outcome of our research, Moso bamboo seeds are suitable substitute candidate for rice and other grain cereals. We found that Fe and Zn concentrations in bamboo seeds are two-fold and 1.7-fold higher than in rice, therefore, consumption of bamboo seeds is an alternative recourse for daily Fe and Zn intake than others such as rice.

A high concentration of Fe and Zn in bamboo seeds does not exactly in accordance with high bioaccessibility and bioavailability of Fe and Zn in human consumption. In our study, following checking bioaccessibility of Fe and Zn, we found that Fe concentrations were decreased from 56.2, 45.6 and 51.1 mg kg^−1^ to 25.3, 25.6, and 25.1 mg kg^−1^, respectively, and Zn concentrations were decreased from 84.9, 80.5 and 71.6 mg kg^−1^ to 18.6, 22.8, and 20.7 mg kg^−1^, respectively (Fig. 2a, c). We also observed that bioavailability of Fe and Zn was decreased. The decreased bioaccessible and bioavailable concentrations of Fe and Zn, compared with the original Fe and Zn concentrations, indicated that some anti-nutritional factors probably play a key role in inhibiting Fe and Zn bioaccessibility and bioavailability in Moso bamboo seeds.

In the past few decades, anti-nutritional factors, including PA, tannins, and fiber have been recognized to inhibit mineral absorption from food consumed by humans^7,29^. For example, PA is the main storage form of phosphorus and minerals in plant seeds, and can considerably inhibit mineral bioavailability^25,30^. In many plant species, such as beans, wheat, and rice, PA has been reported to be a major inhibitor of mineral bioavailability, which decreases Fe and Zn bioavailability in these plants^31–33^. Therefore, in the present study, we first investigated PA concentration in bamboo seeds and rice. We found that PA content in Moso bamboo seeds was significantly lower than in rice (*P* < 0.05), and molar ratios of PA: Fe and PA: Zn were also much lower in bamboo seeds than in rice (Fig. 3a and Table 1). These results indicate that PA in Moso bamboo may not be the key factor that influences the bioaccessibility of Fe and Zn in Moso bamboo seeds. We further investigated the tannins concentrations in Moso bamboo seeds and rice. Different from PA, the tannins concentration in Moso bamboo seeds was significantly higher than in rice (*P* < 0.05). Similar to PA, tannins is able to bind mineral elements by forming insoluble complexes, which renders the minerals less able to be absorbed^34^. Therefore, we presumed that high concentration of tannins in Moso bamboo seeds probably have some level of effect on the Fe and Zn bioaccessibility and bioavailability. These results were further confirmed by PA and tannins concentrations after boiling. After boiling, 80% of the PA was degraded where only 23% of tannins was degraded in bamboo seeds, indicating that tannins in bamboo seeds play a critical role in inhibiting the absorption of Fe and Zn.

The effect of the boiling process on the bioaccessibility and bioavailability of Fe and Zn in bamboo seeds and rice was also investigated. Food processing methods, such as steaming, heating, boiling have different effect on the nutritional quality^25,35^. Eg. Boiling has two different effects on the bioavailability of mineral nutrients. First, boiling can inactivate anti-nutritional compounds and promote the release of mineral nutrients, thus, improving both bioaccessibility and bioavailability of mineral nutrients^6^. In contrast, boiling has been reported to reduce and degrade nutrients, including decreasing mineral content and availability^19,36^. In the present study, we found boiling has a negative effect on bioaccessibility and bioavailability of Fe and Zn in seeds in comparison to uncooked raw seeds (Fig. 2, S1). These results are in good accordance with other studies, for example, in common beans, up to 40% of Fe and Zn were lost during the cooking process and bioaccessibility and bioavailability of Fe and Zn were also reduced^19^.

Combined histochemical staining and LA-ICP-MS approaches indicated that most of the Fe and Zn were localized in embryo and the aleurone layer of the endosperm, and only low levels of Fe and Zn were observed in starchy endosperm. These results suggested that in food processing, it is better to retain embryo and aleurone layer of bamboo seeds to retain more Fe and Zn. This is similar to rice; brown rice (wholegrain rice) contains embryo and the aleurone layer, which contain the majority of the micronutrients. The embryo and aleurone layer are removed to give white rice, which mainly acts as a source of dietary calories and is a poor source of micronutrients, including Fe and Zn^37^. Therefore, increasing numbers of countries are promoting brown rice consumption. When bamboo seeds are consumed as a regular part of the diet, the embryo and the aleurone layer should be retained, similar to the situation with brown rice, to maximize the nutritional benefit.

To prevent Fe and Zn deficiency, artificial efforts to increase levels of Fe and Zn in plants, including plant breeding, transgenic techniques, and agronomic practices, have been investigated to obtain a final food product with a higher amount Fe and Zn content. In the present study, we found that Moso bamboo seeds are a natural food that has high concentrations of Fe and Zn. The quantity of Fe and Zn contained in plant seeds depends on at least two factors: external conditions and internal physiology/inherent factors^14^. For example, climatic conditions, the soil types, availability of nutrients and beneficial microbes in rhizosphere are external factors that can have a great effect on Fe and Zn accumulation in seeds. While physiology/inherent factors, such as the ability to uptake Fe and Zn, root-to-shoot translocation, Fe and Zn distribution and redistribution, and gene expression, directly influence Fe and Zn accumulation in plant seeds. In the present study, we collected bamboo seeds from three different geographical sites where the climate condition is similar, but these sites have a different mean annual precipitation and temperature. While the soil type is classified as a krasnozem but they have a different concentration of available Fe and Zn. The beneficial microbes in rhizosphere were also reported play a crucial role in mineral nutrient uptake in many plant species^38,39^. In bamboo, arbuscular mycorrhizal fungi, bacteria and other microbes were reported to have functional in promoting seedling growth and nutrients uptake^39–41^. However, the exact role of those beneficial microbes in Fe and Zn uptake is still unknown. Similarly, we also have no such data about the difference of the microbial communities of the three different sits in this study. However, form the results we got, no matter what the external factors changes, all the seeds collected from different sits contained much higher Fe and Zn concentrations than rice, which indicated that external factors were probably not the key factors responsible for the high Fe and Zn accumulation in bamboo seeds.

In this study, we thus presumed that physiology/inherent factors may play an important role in Fe and Zn accumulation in bamboo seeds. In many cereal crops, such as rice and wheat, sophisticated Fe and Zn transporter systems have been identified for efficient Fe and Zn transport, which play a critical role in Fe and Zn accumulation^12^. Although to date, these transporters in Moso bamboo are still poorly understood. Since Moso bamboo is diploid, the expression of Fe and Zn transporters may be higher in bamboo than in rice and this may be one of reasons that Moso bamboo is able to accumulate high Fe and Zn concentrations in the seeds. However, further studies are needed to check the exact reasons for high accumulation of Fe and Zn in Moso bamboo seeds.

In conclusion, evaluation of the bioaccessibility and bioavailability of Fe and Zn in Moso bamboo seeds indicated that not only did bamboo seeds contain high concentrations of Fe and Zn but that the Fe and Zn were highly bioaccessible and bioavailable, which indicated that bamboo seeds may be a valuable option as a food for natural Fe and Zn biofortification.

## Methods

### Seed materials and site description

All Moso bamboo (*Phyllostachys edulis*) seeds used in this study were wild-type which were collected from three different geographical sites in Guangxi Province, southwestern China: (1) Haiyang County (25°18’ N, 110°33’ E), which has a mean annual precipitation of 1601 mm and mean annual temperature of 17.5 °C; (2) Fusui County (22°57’ N, 107° 3’ E), which has a mean annual precipitation of 1300 mm and mean annual temperature of 21.7 °C; and (3) Lingchuan County (25°48’ N, 110°07’ E), which has a mean annual precipitation of 1941 mm and mean annual temperature of 18.7°C. The above sampling sites are characterized by a subtropical monsoon climate without any fertilizer input. The soil type is classified as a krasnozem with pH of 4.4–5.6. The soil available Fe and Zn were ranged from 15.9 to 81.3 mg kg^−1^ and 0.8 to 1.9 mg kg^−1^, respectively. The data of available mineral elements are shown in table S1. Rice seeds (*Oryza sativa*, cv Nipponbare) were used as a control.

### Determination of Fe and Zn in seeds

For determining Fe and Zn concentrations, collected seeds were dried at 70 °C in an oven for 3 days. The dried samples were then microwave-digested in a mixture of 3 mL of HNO_3_ and 3 mL of H_2_O_2_ at temperatures up to 180 °C in TFM tubes (Microwave Closed System MAR6, CEM Co., Ltd.). After appropriate dilution, Fe and Zn concentrations in the digested solution were determined by inductively coupled plasma mass spectroscopy (ICP–MS, X series 2; Thermo Scientific).

### Assessment of Fe and Zn bioaccessibility and bioavailability in bamboo seeds by in vitro gastrointestinal digestion

To evaluate bioaccessibility and bioavailability of Fe and Zn in Moso bamboo seeds were investigated, in vitro simulated gastrointestinal digestion protocol was used according to the method described by Coelhoa et al. (2021)^6^ with slight modifications. Briefly, 200 mg of ground raw or cooked (seeds boiled in deionized water for 1.5 h) seeds were placed in 15-mL centrifuge tube. To simulate oral digestion, 200 µL of 1% (w/v) α-amylase solution in pH 6.8 NaHCO_3_ buffer was added to the tube and the solution was mixed well and allowed to stand for a period of time. To simulate gastric digestion, 3 mL of 0.5% (w/v) pepsin solution at pH 1.2 (pH adjusted with concentrated HCl) was added and the mixture was incubated at 37 °C with agitation at 70 rpm. Two hours later, 3 mL of mixed solution of 3% (w/v) pancreatin and 2.5% (w/v) bile salts in pH 7.4 NaHCO_3_ buffer solution were added to the above mixture to simulate the final stage of intestinal digestion. A dialysis membrane (14 kDa; MWCO 3 cm) containing NaHCO_3_ pH 7.4 was inserted into the tube and the mixture was incubated at 37 °C with agitation at 70 rpm for 2 h. Then, the tube was placed in an ice-bath for 15 min to terminate the intestinal enzyme activity. To ensure enzymatic activity, all the reagents used were prepared immediately prior to the digestion protocol. After simulation, the dialysis membrane was removed and washed. The inner solution, which was the bioavailable fraction, was transferred to a glass tube. The gastrointestinal suspension was centrifuged at 4000 rpm for 10 min and the supernatant solution (i.e.; the bioaccessible fraction) was collected in a glass tube. In addition to these samples, blank solutions were prepared with enzymatic and non-enzymatic reagents to correct for possible contamination. Fe and Zn concentrations in the inner and outer solutions were determined by ICP-MS as described above.

### Determination of PA and tannins content in seeds

PA content was determined in accordance with the method of Poblaciones and Rengel (2016)^42^ with slight modification. In brief, 0.5 g of ground seeds was placed in a 15-mL centrifuge tube containing 5.5 mL of 0.01 mol/L HCl. The final mixture was subjected to ultrasonication for 24 min at 40 °C. Following centrifugation at 4000 rpm (for 15 min), the supernatant was diluted with Milli-Q water to 25 mL. Then, 1 mL of diluted supernatant was mixed with 4 mL of Wade reagent [0.03% iron (III) chloride, 0.3% sulfosalicylic acid]. The final solution was then centrifuged at 4000 rpm for 10 min. The absorbance of the supernatant was measured at 500 nm using a spectrophotometer (UV 2600; Shimadzu Corporation). The molar ratios between PA (MW = 660 g/mol) and Fe and Zn were calculated.

For tannins determination, first 0.5 g of ground seeds was placed in a 50-mL centrifuge tube and then 15 mL ethanol (60%) was added and the mixture was subjected to ultrasonication at 40 °C for 30 min. After being filtered with a funnel, the solution was diluted with Milli-Q water to final volume of 50 mL. Next, 1 mL of the supernatant was added to a 15-mL tube and mixture of 5 mL of water and then 0.5 mL of Fulin phenol were added and the final solution was agitated well. After 1 min, mixture of 1.5 mL of 20% NaCO_3_ and 2 mL of water. The mixed solution was kept in the dark for 2 h before the absorbance of the supernatant at 700 nm was measured using a spectrophotometer (UV 2600; Shimadzu Corporation).

### Histochemical staining of seeds

Moso bamboo seeds and rice were soaked in deionized water at 4 °C for 2 days. Then, transverse sections in the root primordium, shoot primordium, and endosperm were cut using a slicer (Fig. 5). For Zn staining, transverse sections were stained with 500 mg/L dithizone methanol solution for 0.5 h, followed by washing three times with deionized water. For Fe staining, transverse sections were immersed in an iron staining solution prepared with a Prussian blue iron stain kit (Solarbio, Hangzhou, China). After 40 min, the sections were washed with deionized water for three times. The stained transverse sections were observed and photographed under a stereo microscope (ZEISS SteREO Discovery.V12).

### Mapping of Fe and Zn deposition in Moso bamboo seeds

Moso bamboo seeds were subjected to laser ablation inductively-coupled plasma mass spectrometry (LA–ICP–MS) (8900; Agilent Technologies) elemental mapping according to the method described by Yamaji and Ma (2019)^43^. Briefly, transverse and longitudinal sections (40 μm) of Moso bamboo seeds were cut with cryotome (HM525, Thermo Fisher Scientific) at –15 °C and transferred onto glass slides and left to dry in freezer (–20 °C) overnight. Then, LA–ICP–MS analysis was performed with LA unit (NWR213; New Wave Research) equipped with an Nd: YAG solid-state laser source. Elemental signals were obtained under no-gas mode by ICP–MS. Time-course ICP–MS output of the raster scanning was converted to elemental images using iQuant2 software. At least three biological replicates of each sample were analyzed and showed similar results.

### Statistical analysis

Data were analyzed using one-way ANOVA, followed by Tukey’s multiple comparison tests. *P* < 0.05 was considered to be statistically significant.

### Reporting summary

Further information on research design is available in the Nature Research Reporting Summary linked to this article.

## Supplementary information

reporting-summary

Supplementary figures
