# Supplementary material for: The potential of bamboo seeds for natural biofortification of dietary zinc and iron
Source: NPJ Sci Food. 2023 Apr 21;7:15. doi: 10.1038/s41538-023-00192-4 (PMC10119318; doi:10.1038/s41538-023-00192-4)
Supplement: Supplementary file 2 — Supplementary figures [file 41538_2023_192_MOESM2_ESM.pdf]

Supplementary Figures/Table

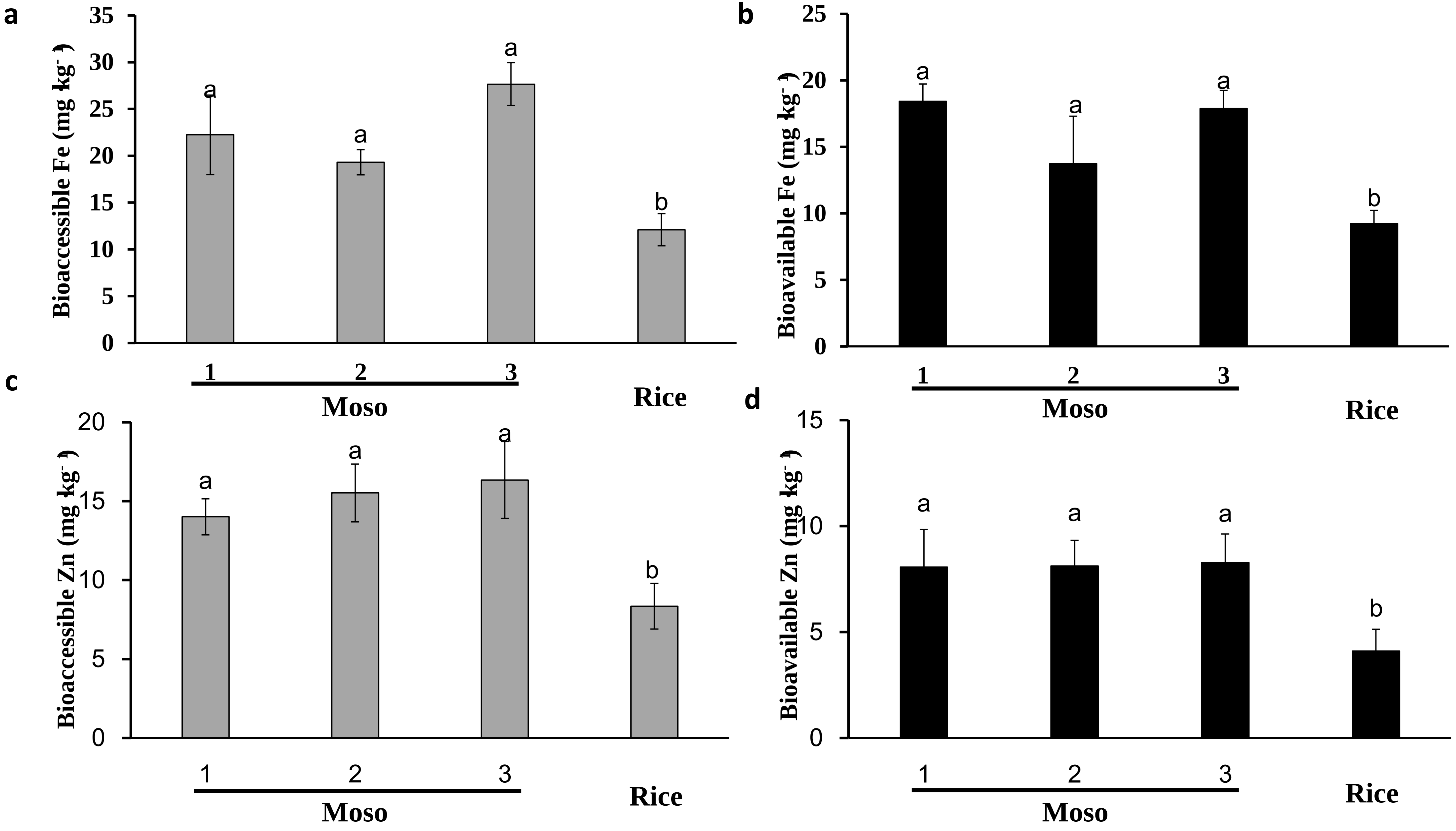

Supplementary Fig.S1 Bioaccessible and bioavailable of Fe and Zn concentration in Moso bamboo seeds and rice after boiling. a, bioaccessible of Fe concentration in Moso bamboo seeds and rice. b, bioavailable of Fe concentration in Moso bamboo seeds and rice. c, bioaccessible of Zn concentration in Moso bamboo seeds and rice. d, bioavailable of Zn concentration in Moso bamboo seeds and rice. Seeds of bamboo and rice were simulated a gastrointestinal digestion in vitro, the Fe and Zn concentration in each fraction was determined by ICP-MS. Data are means ±SD (n=3). Statistical comparison was performed by one-way ANOVA, followed by Tukey's multiple comparison test. Different lower-case letters indicate significant difference at $P<0.05$.

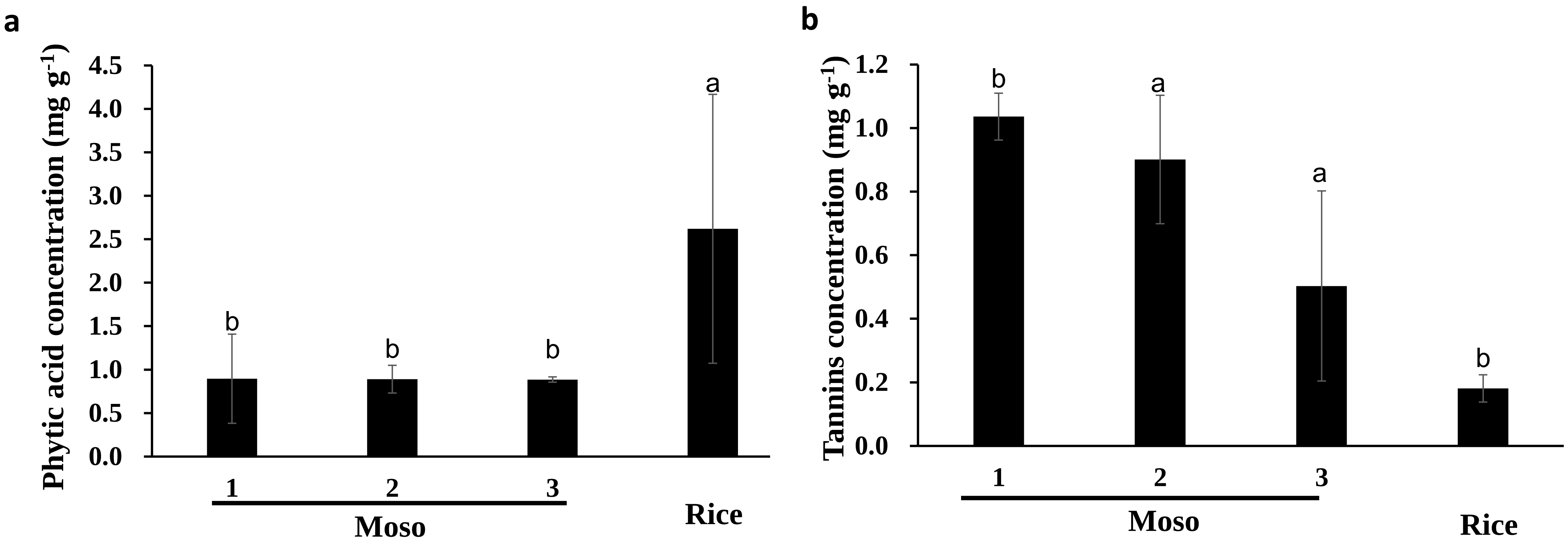

Supplementary Fig.S2. Phytic acid and tannins concentration in Moso bamboo seeds and rice after boiling. a, phytic acid concentration in Moso bamboo seeds and rice. b, tannins concentration in Moso bamboo seeds and rice. Data are means ±SD (n=4). Statistical comparison was performed by one-way ANOVA, followed by Tukey’s multiple comparison test. Different lower-case letters indicate significant difference at $P<0.05$.

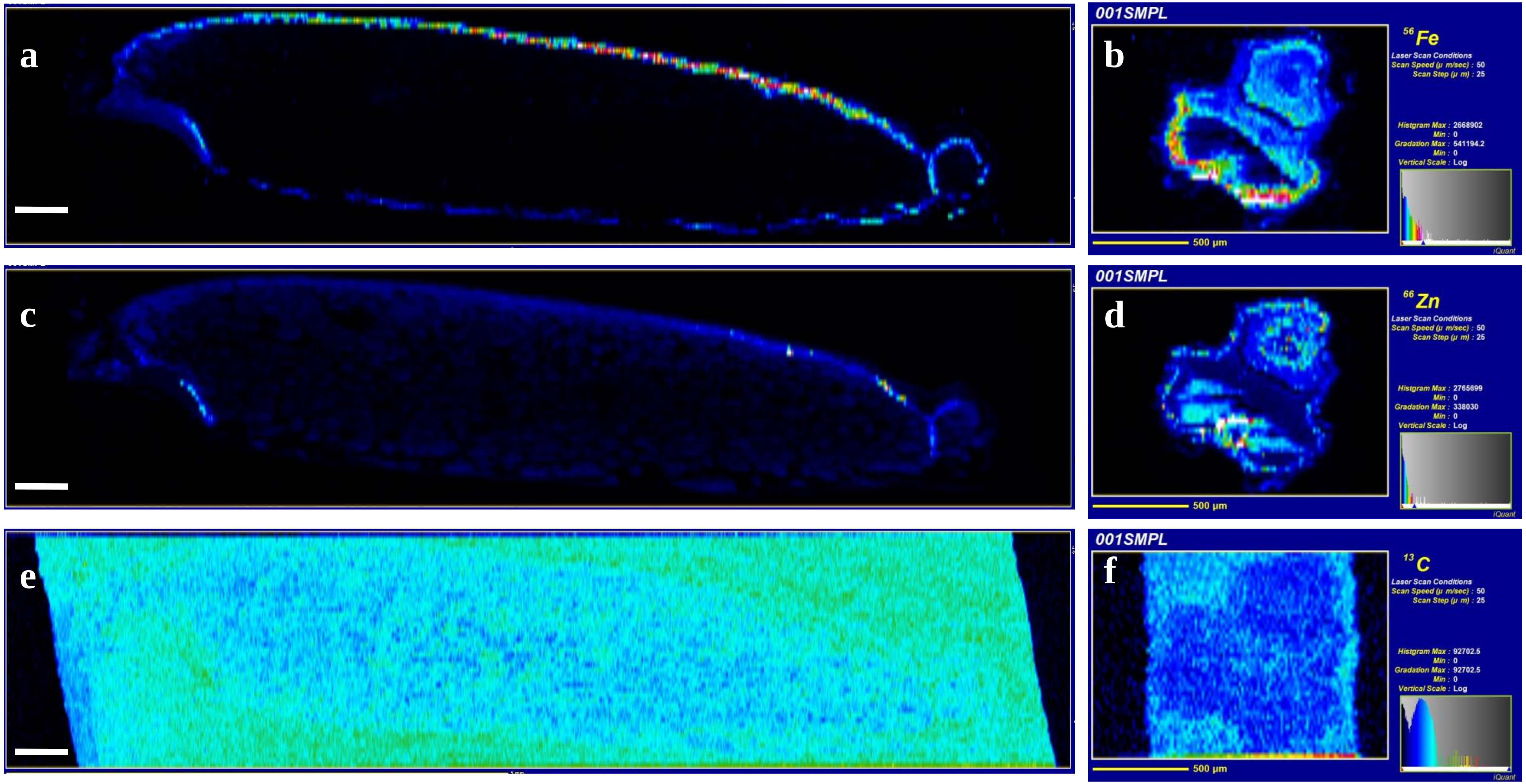

Supplementary Fig.S3. Deposition of Fe, Zn and C in longitudinal and transverse sections of Moso bamboo seed. Longitudinal and transverse sections of Moso bamboo seed were cut with a slicer and elemental imaging was mapped by LA–ICP–MS. The distribution of Fe (a, b), Zn (c, d) and C (e, f) in longitudinal (a, c, e) and transverse sections (b, d, f) of Moso bamboo seeds was presented. Scale bars = 500 μm.

Supplementary Table.S1 available mineral elements of soil collected from three different geographical sites

Sites	Available mineral (mg kg^{-1})									pH
	P	K	Ca	Mg	Fe	Mn	Zn	Cu	B	pH
(1) Haiyang	26.5	103.6	266.8	106.1	15.9	7.7	1.9	0.8	0.39	4.9
(2) Fusui	15.7	65.7	213.6	15.1	81.3	7.1	1.2	0.66	0.37	4.4
(3) Lingchuan	17.4	80.7	580.2	56.1	31.8	13.5	0.8	1.3	0.43	5.6

Data are means ± SD (n=3).
